# Craniofacial Reconstruction Using Rational Cubic Ball Curves

**DOI:** 10.1371/journal.pone.0122854

**Published:** 2015-04-16

**Authors:** Abdul Majeed, Abd Rahni Mt Piah, R. U. Gobithaasan, Zainor Ridzuan Yahya

**Affiliations:** 1 Division of Science and Technology, University of Education, Town Ship Lahore, Pakistan; 2 School of Mathematical Sciences, Universiti Sains Malaysia, 11800 Penang, Malaysia; 3 School of Informatics & Applied Mathematics, University Malaysia Terengganu, 21030 Kuala Terengganu, Malaysia; 4 Institute of Engineering Mathematics, Universiti Malaysia Perlis, Pauh Putra Campus, 02600 Pauh, Perlis; Medical University of South Carolina, UNITED STATES

## Abstract

This paper proposes the reconstruction of craniofacial fracture using rational cubic Ball curve. The idea of choosing Ball curve is based on its robustness of computing efficiency over Bezier curve. The main steps are conversion of Digital Imaging and Communications in Medicine (Dicom) images to binary images, boundary extraction and corner point detection, Ball curve fitting with genetic algorithm and final solution conversion to Dicom format. The last section illustrates a real case of craniofacial reconstruction using the proposed method which clearly indicates the applicability of this method. A Graphical User Interface (GUI) has also been developed for practical application.

## Introduction

Craniofacial refers to the study and treatment of certain congenital malformations or facial injuries. Craniofacial fractures are common with the principal causes by sports-related injuries, gunshot wounds, and motor vehicle accidents. Frequently encountered craniofacial fractures hold certain distinct patterns. Sometimes the fracture is small and at times it makes a large cavity in the skull. A direct surgical reconstruction is complex, time consuming and expensive, where the surgeon relies on personal experience to reconstruct the structure back to its normal contour.

Today, the emerging virtual craniofacial reconstruction employs computer vision technologies which opens its door for mathematicians, engineers, and doctors to visualize, identify and reconstruct the hard/soft tissues using various curves and splines see [1–10] and references therein.

Ball curve was first introduced by Alan Ball at British Aircraft Corporation (BAC) which was featured in BAC Computer Aided Design (CAD) system [11]. It has a number of similar features as the world renowned Bezier curves such as symmetry, satisfies convex hull property, variation diminishing property, coordinate system independence, invariant under affine transformation and interpolates end points.

The distinct advantages of Ball basis functions over Bezier basis functions are twofold. Firstly, a robust algorithm has been developed to evaluate the Ball curve which suits interactive design environment [12–15]. Secondly, generalized Ball basis suits much better in degree elevation and reduction which eases data portability and curve approximation in CAD systems [16, 17]. We have chosen rational Ball curve for the reconstruction of craniofacial due to these reasons. Other advantages include the flexibility, easy shape tweaks by changing control points and extra free parameters which can be used to satisfy design constraints.

This paper proposes the construction of missing portion of traumatised parts of the craniofacial region using rational cubic Ball curves and the final solution is represented in Dicom format for direct application. The next section discusses on the representation of rational cubic Ball basis functions. It is followed by an explanation on boundary extraction and corner detection, and then on genetic algorithm (GA) which is used as an optimization engine to obtain the best fit for derived corner points. An algorithm on craniofacial reconstruction using rational Ball curve is proposed after the explanation of GA configuration. A real case of craniofacial fracture has been chosen to show the applicability of proposed algorithm. These data are obtained from Universiti Sains Malaysia (USM) Health Campus. Matlab has been used to develop a Graphics User Interface (GUI) for craniofacial reconstruction using the proposed algorithm, which can be used by surgeons who may employ this tool without detailed understanding of its mathematical knowledge.

## The Rational Cubic Ball Interpolant

**Fig 1:**
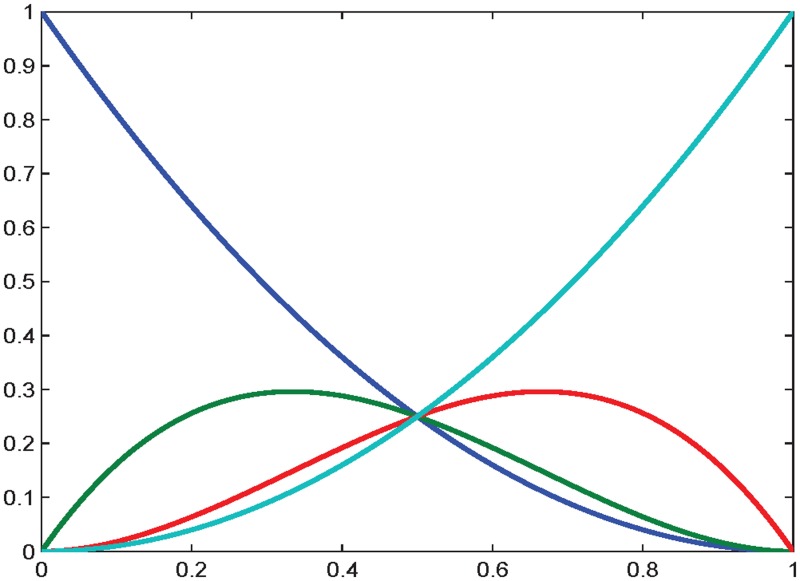
Ball basis functions.

The cubic Ball polynomial basis was first proposed by Ball [11] for CAD systems application. Fig 1 illustrates these functions against its parameter *θ*. The Ball basis functions can be written as

$$\begin{array}{c} S_{0}\left(\theta \right)={\left(1-\theta \right)}^{2}, \end{array}$$

$$\begin{array}{c} S_{1}\left(\theta \right)=2\theta {\left(1-\theta \right)}^{2}, \end{array}$$

$$\begin{array}{c} S_{2}\left(\theta \right)=2\theta^{2}\left(1-\theta \right), \end{array}$$

$$\begin{array}{c} S_{3}\left(\theta \right)=\theta^{2}, \end{array}$$

where

$$\begin{array}{c} \sum_{i=0}^{3}S_{i}\left(\theta \right)=1. \end{array}$$

These functions were further generalized by Said to arbitrary degree [12]. He further proposed a recursive formula to compute Ball curve which were proven to be more efficient than Bezier curves. A rational cubic Ball curve for craniofacial reconstruction is given as follows:

$$\begin{array}{c} s_{i}\left(\theta \right)=\frac{P_{i}\left(\theta \right)}{Q_{i}\left(\theta \right)},i=1,\ldots n-1\;\text{with}\;0\leq \theta \leq 1, \end{array}$$ (1)

where

$$\begin{array}{c} P_{i}\left(\theta \right)=f_{i}{\left(1-\theta \right)}^{2}+V_{i}{\left(1-\theta \right)}^{2}\theta +W_{i}\left(1-\theta \right)\theta^{2}+f_{i+1}\theta^{2}, \end{array}$$

$$\begin{array}{c} Q_{i}\left(\theta \right)={\left(1-\theta \right)}^{2}+a_{i}{\left(1-\theta \right)}^{2}\theta +b_{i}\left(1-\theta \right)\theta^{2}+\theta^{2}, \end{array}$$

and

$$\begin{array}{c} 0=\theta_{0}<\theta_{1}<\theta_{2}<\theta_{3}\ldots .<\theta_{n}=1, \end{array}$$

$$\begin{array}{c} V_{i}=a_{i}f_{i}+h_{i}d_{i}, \end{array}$$

$$\begin{array}{c} W_{i}=b_{i}f_{i+1}-h_{i}d_{i+1}. \end{array}$$

Eq (1) satisfies the following conditions

$$\begin{array}{c} \left\{ \begin{array}{c} s_{i}\left(\theta_{0}\right)=f_{i},\;s_{i}\left(\theta_{n}\right)=f_{i+1},\\ s_{i}^{\prime }\left(\theta_{0}\right)=d_{i},\;s_{i}^{\prime }\left(\theta_{n}\right)=d_{i+1}. \end{array}\right. \end{array}$$ (2)

*a*_*i*_, *b*_*i*_ are free parameters, *f*_*i*_, *f*_*i*+1_ are the endpoints of each segment, and *d*_*i*_, *d*_*i*+1_ are unit tangent vectors at *f*_*i*_ and *f*_*i*+1_, respectively.

### Boundary Extraction and Corner Detection

The first step on craniofacial reconstruction is to identify the boundary of missing part from a scanned image which is in Dicom format. The boundary of the original image is obtained using mathematical morphology defined by *β*(*A*) = *A* − (*A*Θ*B*) where *A* is the set of all black pixel, *B* is the 3×3 structuring element and *β*(*A*) is the boundary of set *A*. Θ and —represent the erosion and difference operator, respectively. The corner points are used to divide the boundaries into smaller segments. This paper employs Sarfraz et al.’s method [18] to identify the corner points.

### Parameterization

Chord length parameterization is used to evaluate the values of *θ*_*i*_ related to points *f*_*i*_ = *D*_*i*_, where *D*_*i*_ represent the data points of segments,

$$\begin{array}{c} \left\{ \begin{array}{c} \theta_{0}=0,\\ \theta_{k}=\frac{\sum_{i=1}^{k}\left|D_{i}-D_{i+1}\right|}{\sum_{i=1}^{n}\left|D_{i}-D_{i+1}\right|}\;1\leq k\leq n-1,\\ \theta_{n}=1. \end{array}\right. \end{array}$$ (3)

It can be observed that *θ*_*i*_ is a normalized form and varies from 0 to 1 with *h*_*i*_ = *θ*_*i*+1_ − *θ*_*i*_. Hence, $h=\sum_{i=1}^{n-1}h_{i}=1$.

### Tangent Vectors

In general, the tangent vectors *d*_*i*_ at *f*_*i*_ are defined as follows:

For open curve,

$$\begin{array}{c} \left\{ \begin{array}{c} d_{0}=2\left(f_{1}-f_{0}\right)-\left(f_{2}-f_{0}\right)/2,\\ d_{n}=2\left(f_{n}-f_{n-1}\right)-\left(f_{n}-f_{n-2}\right)/2,\\ d_{i}=a_{i}^{*}\left(f_{i}-f_{i-1}\right)-\left(1-a_{i}^{*}\right)\left(f_{i+1}-f_{i}\right), \end{array}\right. \end{array}$$

for *i* = 1, .…, *n* − 1.

For close curve,

$$\begin{array}{c} \left\{ \begin{array}{c} f_{-1}=f_{n-1},\;f_{n+1}=f_{1},\\ d_{i}=a_{i}^{*}\left(f_{i}-f_{i-1}\right)-\left(1-a_{i}^{*}\right)\left(f_{i+1}-f_{i}\right), \end{array}\right. \end{array}$$

for *i* = 1, .…, *n* − 1.

where

$$\begin{array}{c} a_{i}^{*}=\frac{|f_{i+1}-f_{i}|}{|f_{i+1}-f_{i}\left|+\right|f_{i}-f_{i-1}|},\;i=0,1,\ldots n. \end{array}$$

We use both open and closed curves. To reconstruct the complete skull we use closed curves and to reconstruct curves of the missing parts for each slice, we use open curves.

### Normalized Mean Squares Error

A normalized mean squares error is used as cost function which is employed to find the errors of free parameters of rational Ball interpolant. The normalized mean squares error is computed by

$$\begin{array}{c} E^{2}=\frac{\sum |s_{i}\left(\theta \right)-D_{i}{|}^{2}}{\sum |D_{i}{|}^{2}}, \end{array}$$ (4)

where *D*_*i*_ represents the pixel values of the segments and *θ* is the parameterized chord length. The goal is to find the values of unknown parameters *a*_*i*_ and *b*_*i*_ in Eq (1) in order to minimize the normalized mean squares error. GA is used to optimize the value of *a*_*i*_ and *b*_*i*_ for craniofacial reconstruction.

### Genetic Algorithm

Once the boundaries and corners are identified, the next step is to fit rational cubic Ball curves to reconstruct the missing craniofacial parts. For this we have to minimize Eq (4) for best fitting rational Ball curve. Two free parameters *a*_*i*_ and *b*_*i*_ are involved in Eq (4). We use a continuous genetic algorithm (GA) as an engine to optimize free parameters *a*_*i*_ and *b*_*i*_ to obtain a suitable fit from given boundaries and corner points.

The GA employed in this study is similar to binary GA where the primary difference is variables are no longer represented by bits of zeros and ones. We start the GA process on *E* ^2^ by defining a chromosome as an array of variables to be optimized. Since there are two variables for our interpolant, the chromosome is represented as an array of 1×2 elements i.e *chromosome* = [*a*_*i*_, *b*_*i*_], *i* = 1, … *n*.

The GA is an optimization engine which must be constrained to explore a reasonable region of variable space. Sometimes this is done by imposing a constraint on the interpolant. For example, if an initial search region is unknown, then we define a suitable search space to start the GA process. It is then followed by stating an initial population of chromosome; a normal practise is the initial population is varied from 2*k* to 4*k*, where *k* is the number of chromosomes. In our case, initial population size is set to be 8. The chromosomes are now ready for evaluation and selection of suitable interpolant.

In the next step, we select chromosomes in the initial population which fits enough to survive and possibly reproduce a better offspring in the next generation. For this, the cost and its associated chromosomes are ranked from lowest to highest. Only the top 50% of population with lowest cost will be selected for crossover which are represented as new set of parents. This process of natural selection occurs at each iteration of the algorithm.

The simplest method for crossover is to choose one or more points in the selected chromosomes to produce various solutions. The variables at these points are merely swapped between the two parents. Mutation is the final step where we change some chromosome values randomly within the search space; usually the mutation rate is set to 20%. This process continues iteratively for a given number of generations or until a result obtained is less than a defined value.

### Curves to Dicom Format

After fitting the missing part with rational Ball curves of each Dicom image, the next step is to convert the missing part into a Dicom format. For this, we take the corner points of both curves and then join the initial and final points by using the following linear form:

$$\begin{array}{c} C_{i}=\left(1-t\right)A_{i}+tB_{i},\;i=0,1 \end{array}$$

where *A*_*i*_ and *B*_*i*_ are initial and final points of curves, and *t* varies from 0 to 1. Our next step is to define a black image (all zero values) of the same size as the original Dicom image slices. The fitted rational Ball curve is then converted into white form by equaling this data to 1. Finally, we use Matlab command *imfill* to fill the area bounded by the curves. To note, ezDicom is a software which is used to read the constructed Dicom image.

### Graphical user interface(GUI)

A graphical user interface (GUI) is graphical display which is used to carry out interactive tasks and consists on one or more windows having controls. These are called components. This interface eases out user from creating and executing the commands in order to complete tasks. In contrast to coding programs GUI user does not have to go into details to understand the procedure of execution of commands. This interface include menu, start and stop buttons, boxes etc.

Each control, and the GUI itself, has one or more user written routines (executable MATLAB code) known as callbacks, named for the fact that they call back to MATLAB to ask it to do things. The execution of each callback is triggered by a particular user action such as pressing a screen button, clicking a mouse button, selecting a menu item, typing a string or a numeric value, or passing the cursor over a component. The GUI then responds to these events. In this article GUI is used to control the curve by using free parameters. The input is the end points of missing part depending on the users. The free parameters allow the user to control the curve.

### Proposed Algorithm

This section simplifies the algorithm for craniofacial fracture reconstruction.

**Fig 2:**
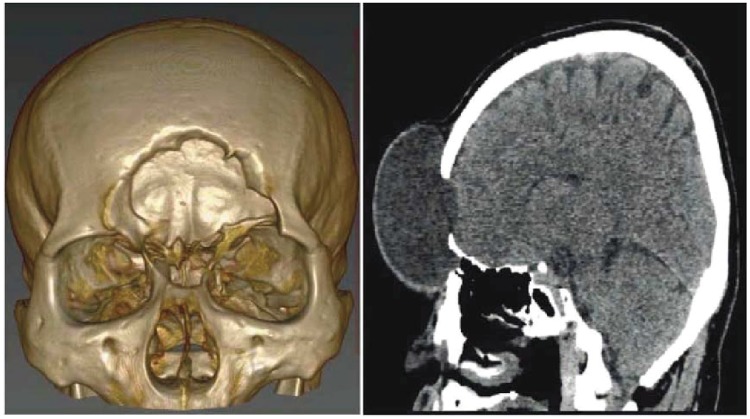
3D slicer image of a patient with head injury.

**Fig 3:**
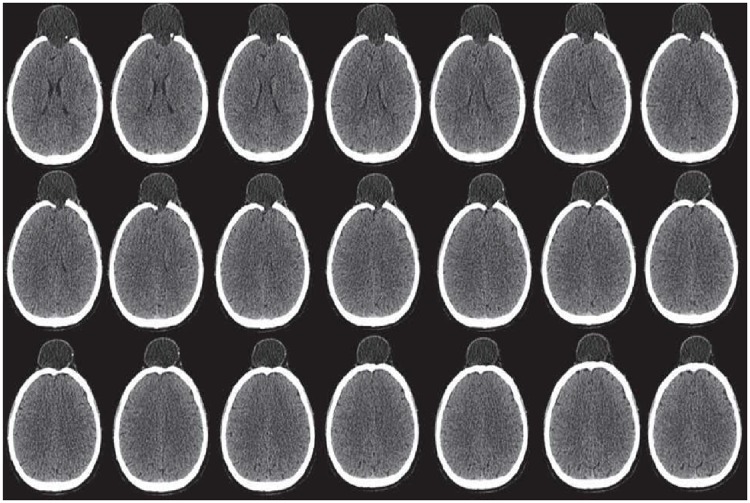
CT scanned images of patient with head injury.

**Fig 4:**
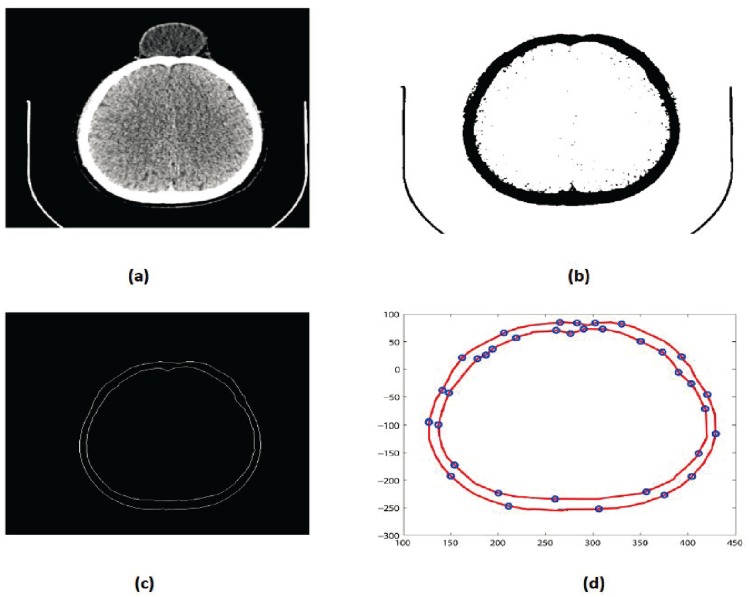
Reconstructed image of CT scan of Dicom slice 176. (a) Original image (b) Binary image (c) Boundary (d)Reconstructed boundary using rational cubic Ball

**Fig 5:**
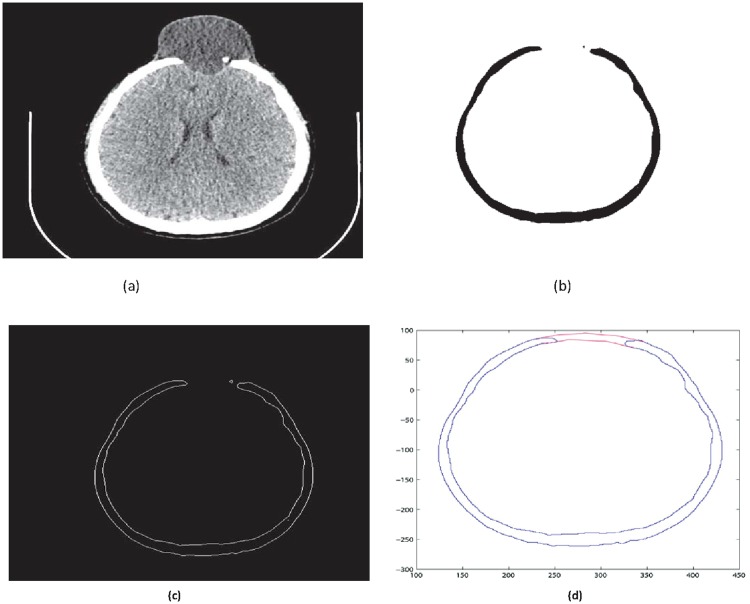
CT scanned data of slice 160. (a)Original image (b) Binary image (c) Boundary (d)Reconstructed missing part using rational cubic Ball

1. Input: CT scan image in Dicom format. (case study: A patient with head injury for craniofacial fracture reconstruction as shown in Figs 2 and 3)
2. Boundaries are divided into segments after obtaining corner points. The blue dots in Fig 4d represent the corner points
3. Each segment is fitted using rational Ball interpolant where the unknown parameters *a*_*i*_ and *b*_*i*_ in Eq (1) are optimized using genetic algorithm. We used sixteen segments for the construction of outer curve of slice 176 in Fig 4d and twenty segments for inner curve shown in Fig 4d
4. Errors are calculated using normalized mean squares equation for all segments of both inner and outer curves of slice 176 see in Table 1
5. Steps 3 and 4 are repeated until a desired solution is obtained
6. The CT scan data used in this case has 189 slices out of these 55 slices have a fracture part
7. Reconstruct the missing part for each Dicom slice separately after converting the image into binary and boundary form Fig 5b and 5c
8. Finally, convert the area enclosed by the curves into Dicom format using Matlab, and ezDicom software is used to visualize the constructed Dicom image
9. GUI is used to construct the missing part with out understanding the mathematics behind

**Table 1:** Slice 176 errors from left to right.

1.420e-06	1.27e-05	3.99e-06	1.68e-07	4.504e-06	7.6055e-06
1.215e-05	1.053e-05	9.963e-06	1.012e-07	6.87e-06	4.85e-06
5.95e-06	5.25e-06	4.59e-06	5.788e-06	3.12e-06	2.96e-0-6
3.38e-06	3.033e-06	3.88e-06	1.356e-07	4.36e-06	3.07e-06
4.65e-06	2.05e-06	3.059e-06	2.76e-06	1.98e-06	9.91e-07
1.3369e-06	1.685e-06	7.41e-07	1.336e-06	1.7911e-06	3.26e-06

## Results and Discussion

### Case Study: A Patient With Head Injury

**Fig 6:**
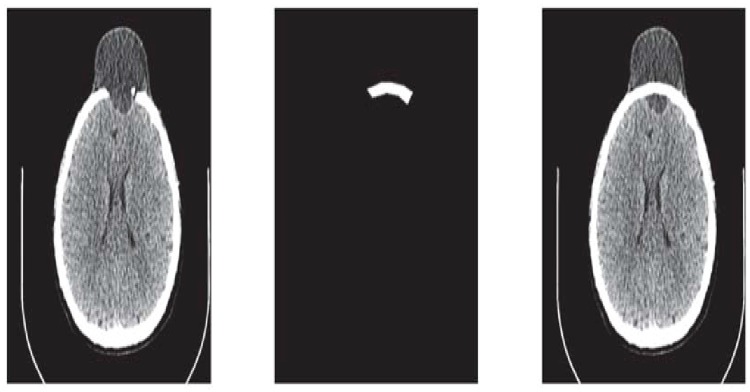
Missing data in Dicom format of slice 160.

**Fig 7:**
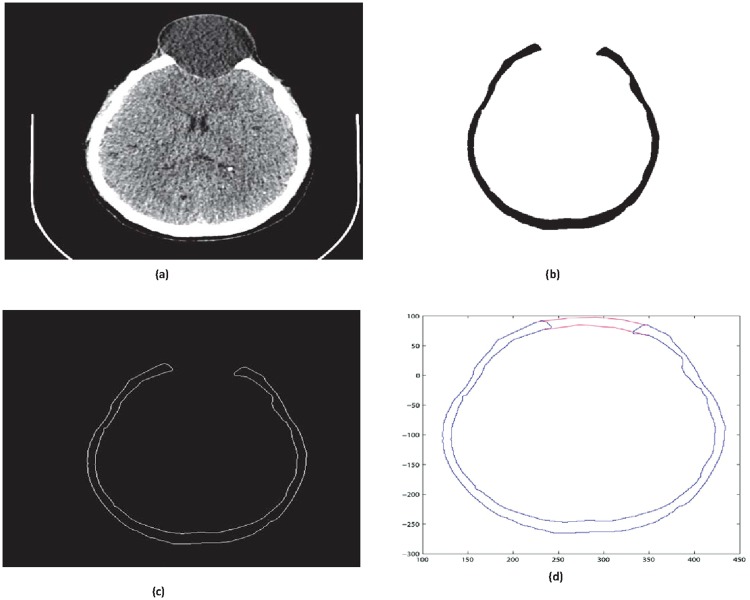
CT scanned data of slice 147. (a)Original image (b) Binary image (c) Boundary (d)Reconstructed missing part using rational cubic Ball

**Fig 8:**
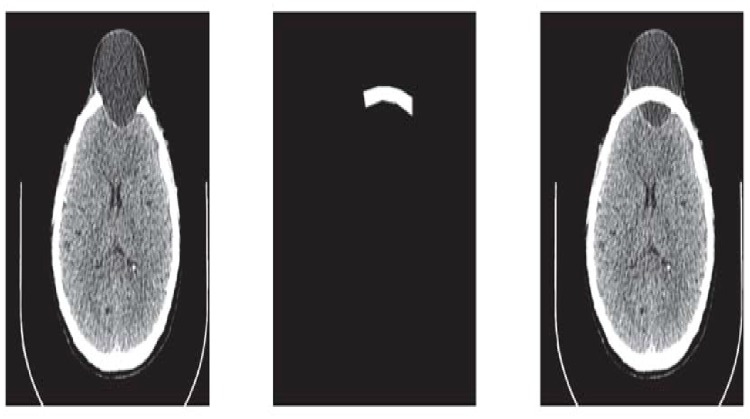
Missing data in Dicom format of slice 147.

**Fig 9:**
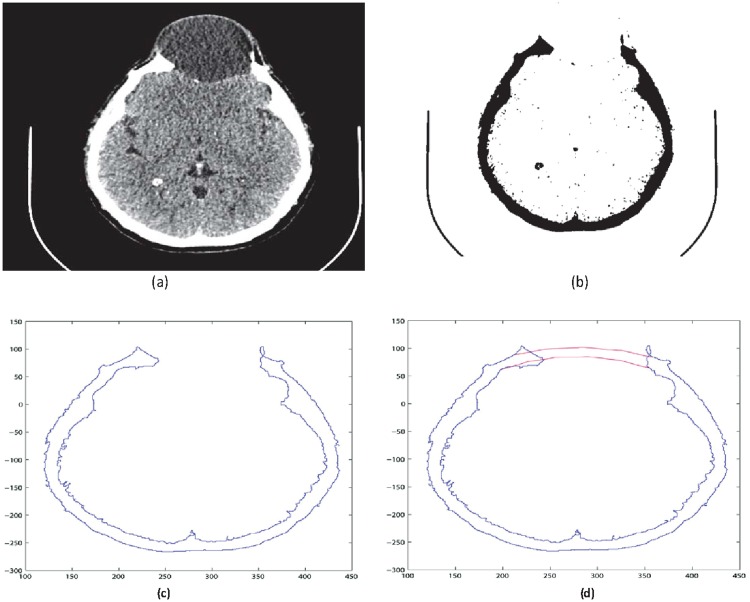
CT scanned data of slice 131. (a)Original image (b) Binary image (c) Boundary (d)Reconstructed missing part using rational cubic Ball

**Fig 10:**
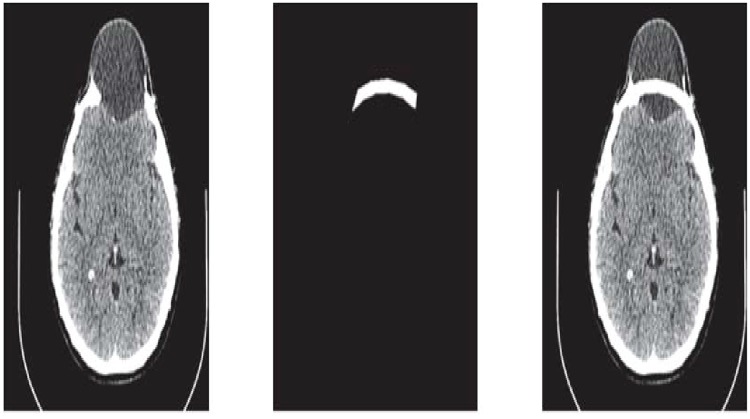
Missing data in Dicom format of slice 131.

**Fig 11:**
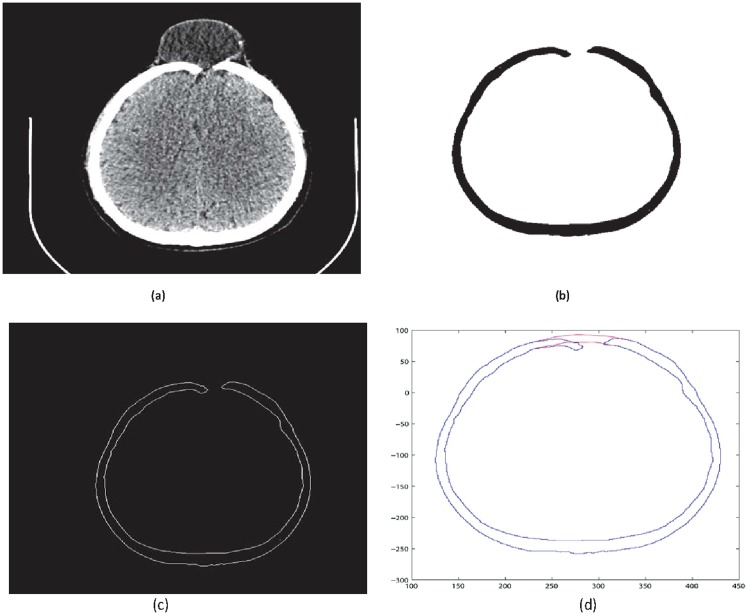
CT scanned data of slice 171. (a)Original image (b) Binary image (c) Boundary (d)Reconstructed missing part using rational cubic Ball

**Fig 12:**
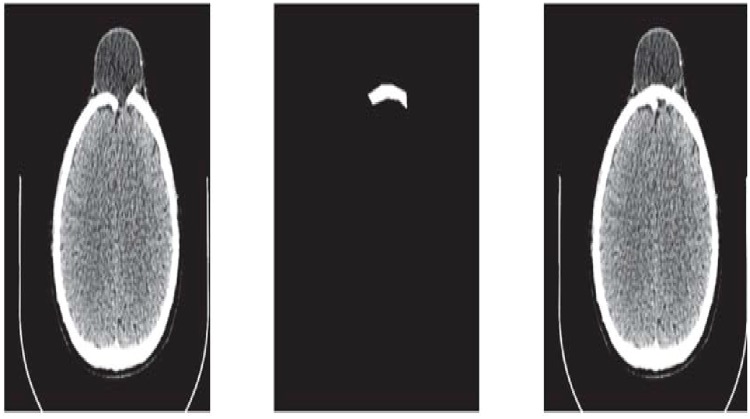
Missing data in Dicom format of slice 171.

**Fig 13:**
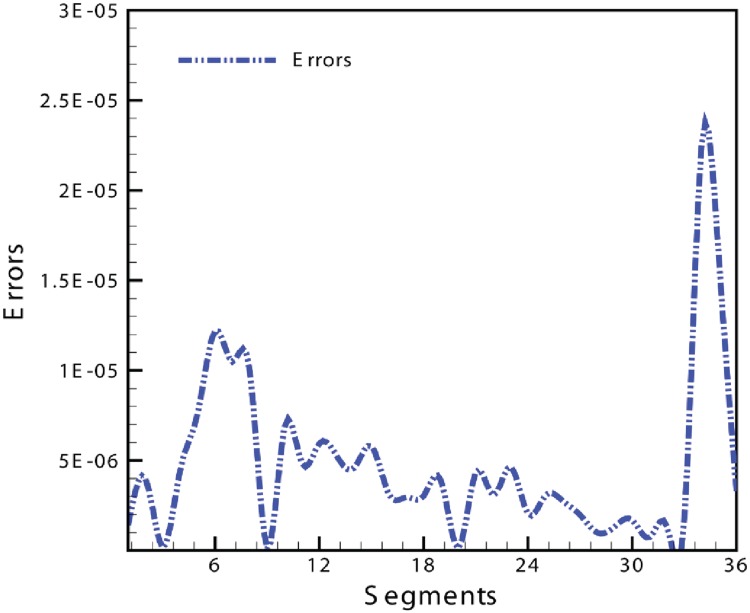
Graph of the normalized mean squares error of slice 176.

**Fig 14:**
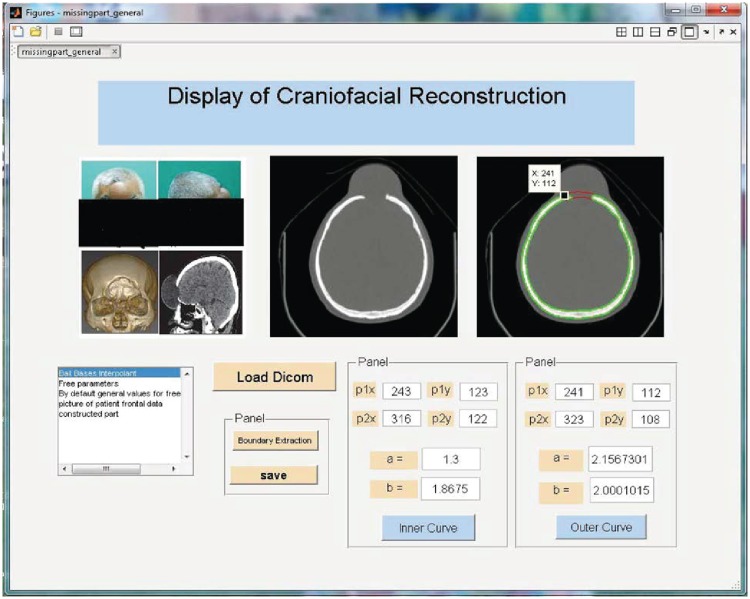
GUI display of craniofacial reconstruction.

This section illustrates an example of application of the proposed algorithm. We have constructed the inner and outer curve for all CT scan slices using rational Ball interpolant. Fig 5a is the original CT scan of slice 160. To construct the traumatized part, we first convert the image to its binary form Fig 5b, then we extracts the boundary of skull shown in Fig 5c using mathematical morphology. Fig 5d shows the reconstructed inner and outer curves. Once a satisfactory result is obtained from GA optimization, we convert the area between two curves into Dicom format as shown in Fig 6. The first image of Fig 6 shows a given Dicom slice of 160, the second image is the missing part obtained from the proposed algorithm in Dicom and third image is the combination of first two images, which is the required Dicom solution. This process is repeated for various CT scan slices as shown in Figs 7–12. The given Dicom data for skull are in sequence and consistent for different frames or contours. The constructed curves of the missing parts using rational cubic Ball interpolant will be consistent between different frames since these curves obey convex hull property, variation diminishing property (VDP), independent of coordinate system and satisfy the end point conditions. By convex hull property all constructed curves will lie within the convex hull of control polygon. Fig 13 represents the graph of normalized mean squares error of slice 176. Fig 14 is the display of Graphical User Interface (GUI).

## Conclusion

This paper proposes craniofacial reconstruction using cubic rational Ball interpolant. A patient with head injury as a case study is illustrated to show its applicability. The computed errors and final outputs indicate that the proposed interpolant is suitable for solving craniofacial reconstruction problem. In addition, a user friendly GUI is developed which surgeons may use for practical applications.
